# Associations of Participation in Organized Sport and Self-Organized Physical Activity in Relation to Physical Activity Level Among Adolescents

**DOI:** 10.3389/fpubh.2019.00129

**Published:** 2019-05-24

**Authors:** Pål Lagestad, Hilde Mikalsen, Laura Suominen Ingulfsvann, Idar Lyngstad, Camilla Sandvik

**Affiliations:** Faculty of Teacher Education and Arts, Nord University, Levanger, Norway

**Keywords:** adolescence, physical activity level, organized sport, self-organized physical activity, health

## Abstract

Although physical activity level (PAL) is positively correlated with adolescents' health, many adolescents do not fulfill recommendations for physical activity. This study examines the associations of organized sport and self-organized physical activity, with PAL among adolescents. Participants were 301 adolescents (12–13 year-olds). The adolescents wore accelerometers for 1 week according to international standards, and reported their participation in organized sport and self-organized physical activity in a questionnaire. The results showed that the level of participation in organized sport was positively associated with the adolescents' total PAL, while there was no significant association between time spent in self-organized physical activity and adolescents' daily minutes of moderate and vigorous physical activity. In addition, boys who participated <3 h per week (or not at all) in organized sport stood out with the lowest fulfillment of recommended PAL. Our findings underline the critical importance of getting adolescents, especially boys, to participate in organized sport and not to drop out from organized sport during adolescence.

## Introduction

The health benefits of having a high physical activity level (PAL) in childhood and youth are well-established (1–12). The benefits of regular physical activity appear early in life and include positive changes in adiposity, skeletal health, psychological health, and cardiorespiratory fitness (7).

However, a major concern is that PAL seems to decrease dramatically over time among adolescents in Norway, as well as in other countries (13–17). In a new study following adolescents' activity level from the age of 14 to the age of 19 with annual measures, accelerometer-measured moderate and/or vigorous physical activity levels declined from 67 min per day at the age of 14, to 24 min per day at the age of 19, i.e., a decrease during the period close to linear (15).

According to current international recommendations for physical activity, Norwegian children should accumulate at least 60 min of moderate to vigorous-intensity physical activity (MVPA) daily (18–20). The most recent Norwegian national monitoring of PAL among children and adolescents occurred in 2011 (21). This study showed that while 69.8% of 9-year-old girls and 86.2% of 9-year-old boys met the recommendations (as measured objectively by accelerometers), the proportions were only 43.2 and 58.1% for 15-year-old girls and boys, respectively.

We argue that, in general, there are two main ways for adolescents to achieve physical activity recommendations: during organized sport and/or during self-organized physical activity. Previous research has reported positive associations between participation in organized sport and PAL or cardiorespiratory fitness (1, 13, 14, 22–26). Bélanger et al. (13) found a higher number of total MVPA sessions per week among adolescents participating in organized physical activities, compared to those not involved in organized physical activities. Dalene et al. (14) reported a positive association between time spent in organized sport and exercise, and levels of MVPA. Marques et al. (24) found a statistically significant relationship between participation in organized sport and rate of fulfilling physical activity guidelines among boys, but not among girls. In a study among adolescents aged 11–13 years, Pearce et al. (27) reported that the adolescents spent few minutes per day in organized physical activity, and that these periods contributed little toward daily minutes of MVPA. However, self-organized outdoor leisure time was correlated with an increase in total daily MVPA of almost twice that of self-organized indoor leisure time.

Previous research has pointed toward a positive association between participation in organized sport and PAL. However, many of these studies did not examine the importance of different levels of participation in organized sport according to PAL and fulfillment of the recommendation of 60 min of MVPA. Furthermore, to the best of our knowledge, no studies have yet examined the importance of different levels of participation in self-organized physical activity, according to PAL and fulfillment of the recommendation of 60 min of MVPA. Therefore, this study will investigate the importance of participation in organized sport and self-organized physical activity during leisure time at different levels, respectively, in relation to PAL and fulfillment of physical activity recommendations among adolescents. Within this approach, the results of previous research also highlight the importance of studying gender differences (21).

## Materials and Methods

### Design

Objective measures of physical activity (accelerometer data) and self-reported data about participation in organized sport and self-organized physical activity were collected. The study was approved by the Norwegian Center for Research Data (NSD). Parents and youths both provided written informed consent to participate in the study.

### Subjects

Two medium-sized Norwegian municipalities (~15–22,000 inhabitants) with 18 lower secondary schools, with a total of 416 12–13 year-old adolescents (born in 2004), were selected for the study. Of these, 301 participants (155 girls and 146 boys) had valid accelerometer and questionnaire data, comprising a response rate of 72.4%. Of the total 301 participants, 187 attended schools close to a city, while 114 attended schools in rural areas. The sample, consisting of adolescents who agreed to participate, is considered random according to the available population in the two municipalities (28, 29).

### Procedures

The ActiGraph GT1M accelerometer was used to measure the adolescents' PAL (minutes of moderate-to-vigorous physical activity [MVPA]). According to the procedures of Norwegian population studies of adolescents (21), subjects wore the accelerometer on a belt placed on the right hip for 1 week (the total data-material were collected within the period of 15 April to 15 May, 2017), removing it only when going into water and sleeping at night.

After 1 week, the accelerometers were collected, and data were downloaded into the ActiLife software programme (ActiGraph) and analyzed. A 10-s epoch was used (21). According to Kolle et al. (21), missing data were defined as a period of 20 continuous minutes or more with no counts. All activity during the night (2400–0600 h) was deleted based on the same test protocol. Furthermore, each day had to include at least 480 min of recorded activity to be considered valid, and each adolescent had to have at least 2 valid days to be included in the analyses. This combination has previously been shown to provide a reliable estimate of children's habitual physical activity (PA) (30). The cut-off for moderate intensity was set at 2,000 counts, according to the test protocol of Kolle et al. (21).

During the same week as the accelerometer data were collected, the study sample answered a questionnaire that included the following questions; “How many hours per week are you training or competing in organized sport at a level that you become at least moderately out-of-breath and perspire?,” and “How many hours per week are you physically active outside of organized sport and physical education at a level that you become at least moderately out-of-breath and perspire? The first item was termed “organized sport,” while the second item was termed “self-organized physical activity.” The response options of these items were: “never,” “1–2 h per week,” “3–4 h per week,” “5–7 h per week,” “8–10 h per week,” and “11 h or more per week.” These questions are used in previous research (31). Such single-item measures have also shown to be valid screening tools to determine whether respondents are sufficiently active to benefit their health (50).

### Statistical Analyses

Chi square tests were used to examine differences between the number of girls and boys who participated in both organized sport and self-organized physical activity. To identify differences in the objectively measured PAL (MVPA), according to different levels of participation in both organized sport and self-organized physical activity among boys and girls, different combinations of participation in organized sport and self-organized physical activity, and interactions between sex and physical activity level, we used univariate analysis of variance (two-way ANOVA). Moreover, if interactions of sex were detected, one-way ANOVA was conducted to identify differences between boys and girls at the six activity levels. One-way ANOVA was also utilized to identify differences according to different levels of participation among boys and girls separately, as a follow-up test if there was a main effect of participation level in both organized sport and self-organized physical activity. The effect size was evaluated with $\eta_{\text{p}}^{2}$ (partial eta-squared), where 0.01 < η^2^ < 0.06 indicates a small effect; 0.06 < η^2^ < 0.14, a medium effect; and η^2^ > 0.14, a large effect (32). If the analyses revealed significant differences, we performed *post-hoc* tests with Bonferroni corrections.

To identify differences in the proportions of adolescents meeting the recommended level of physical activity (60 min MVPA daily) according to different levels of participation in both organized sport and self-organized physical activity, and different combinations of participation, we used the Kruskal–Wallis H test (non-parametric test), with Mann–Whitney *U*-test with Bonferroni corrections, as follow-up tests comparing each of the six activity levels. Mann–Whitney *U*-test was also used to examine sex differences in fulfillment of recommended levels of physical activity. The level for significance was set at *p* < 0.05. The results are presented by numbers, means, and standard deviations. Statistical analyses were performed with SPSS, version 24.0 (IBM, Armonk, NY, U.S.A.).

## Results

Table 1 shows that the number of girls and boys participating at different levels in organized sport did not vary significantly (*x*^2^ = 2.3, *p* = 0.810). However, while approximately half of the adolescents participated between 5 and 10 h per week, only 15 adolescents (5%) participated in organized sport 11 h or more per week.

**Table 1:** Number of girls and boys participating at different levels in organized sport.

**Activity level**	**Girls**	**Boys**	**Total**
No participation	22	21	43
1–2 h per week	17	11	28
3–4 h per week	23	18	41
5–7 h per week	53	53	106
8–10 h per week	33	35	68
11 h or more per week	6	9	15
Total	154	147	301

Table 2 shows that the number of girls and boys participating at different levels in self-organized physical activity also did not vary significantly (*x*^2^ = 9.6, *p* = 0.103). However, while approximately half of the adolescents participated between 5 and 10 h per week, only 9 adolescents (3%) participated in self-organized physical activity 11 h or more per week. The participation pattern seems very much the same in self-organized physical activity as in organized sport.

**Table 2:** Number of girls and boys participating at different levels in self-organized physical activity.

**Activity level**	**Girls**	**Boys**	**Total**
No participation	13	26	39
1–2 h per week	40	31	71
3–4 h per week	57	42	99
5–7 h per week	28	29	57
8–10 h per week	11	15	26
11 h or more per week	6	3	9
Total	155	146	301

**Table 3:** Number of girls and boys participating at different levels in both organized sport and self-organized physical activity.

**Group number**	**Group combination**		
	**Organized sport**	**Self-organized physical activity**	**N**
1	No participation	No participation	3
2	No participation	1–2 h per week	6
3	No participation	3–4 h per week	15
4	No participation	5–7 h per week	12
5	No participation	8–10 h per week	3
6	No participation	11 h per week or more	4
7	1–2 h per week	No participation	4
8	1–2 h per week	1–2 h per week	9
9	1–2 h per week	3–4 h per week	5
10	1–2 h per week	5–7 h per week	9
11	1–2 h per week	8–10 h per week	1
12	1–2 h per week	11 h per week or more	1
13	3–4 h per week	No participation	6
14	3–4 h per week	1–2 h per week	18
15	3–4 h per week	3–4 h per week	12
16	3–4 h per week	5–7 h per week	4
17	3–4 h per week	8–10 h per week	1
18	3–4 h per week	11 h per week or more	1
19	5–7 h per week	No participation	17
20	5–7 h per week	1–2 h per week	22
21	5–7 h per week	3–4 h per week	38
22	5–7 h per week	5–7 h per week	15
23	5–7 h per week	8–10 h per week	12
24	5–7 h per week	11 h per week or more	1
25	8–10 h per week	No participation	8
26	8–10 h per week	1–2 h per week	15
27	8–10 h per week	3–4 h per week	23
28	8–10 h per week	5–7 h per week	14
29	8–10 h per week	8–10 h per week	7
30	8–10 h per week	11 h per week or more	1
31	11 h per week or more	No participation	1
32	11 h per week or more	1–2 h per week	8
33	11 h per week or more	3–4 h per week	2
34	11 h per week or more	5–7 h per week	1
35	11 h per week or more	8–10 h per week	2
36	11 h per week or more	11 h per week or more	1

Table 2 shows that the number of girls and boys participating in different levels in both organized sport and self-organized physical activity, varied a lot, with most adolescents participating 5–7 h per week in organized sport and 3–4 h per week in self-organized physical activity.

**Figure 1:**
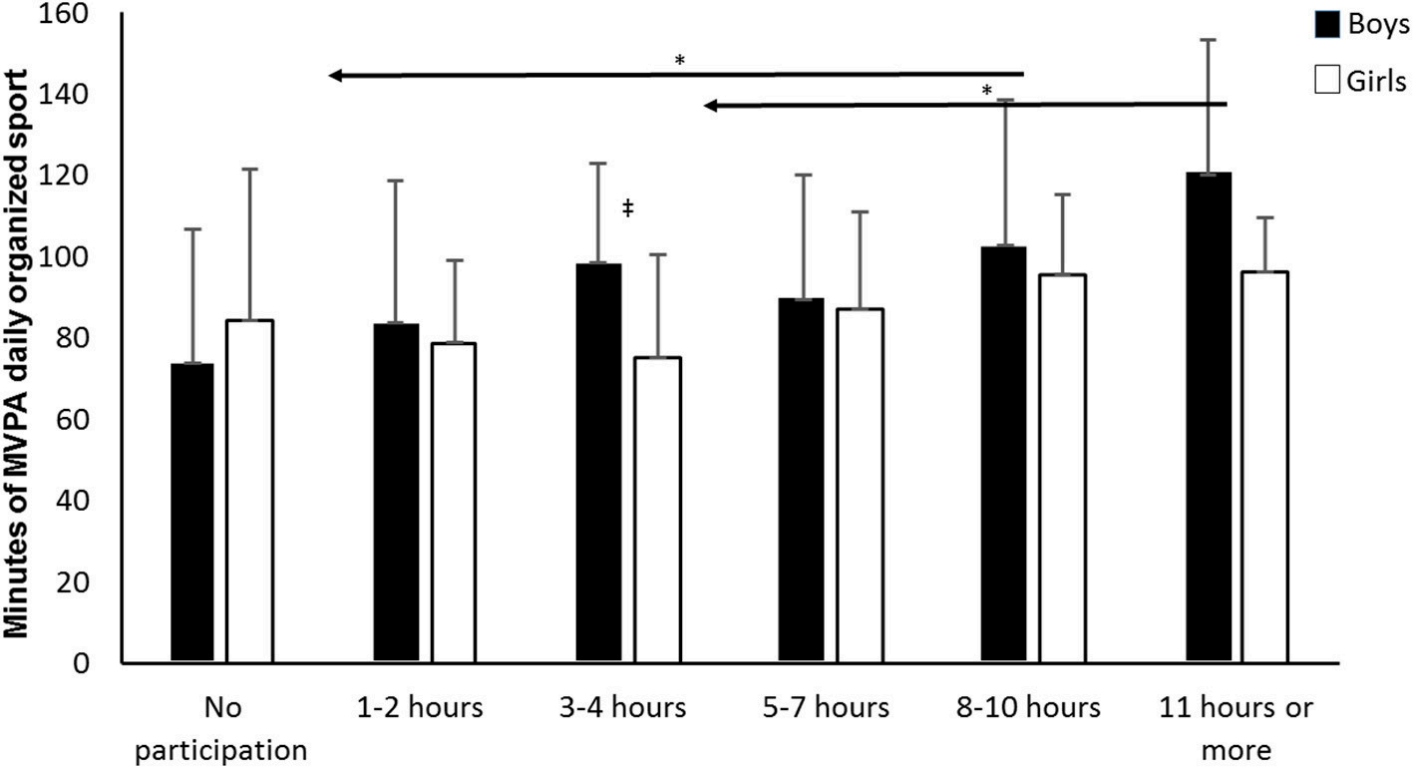
Differences in MVPA level among girls and boys according to increasing participation in organized sport. ^*^Indicates a significant decrease in PAL between these groups and the groups left of the arrow at a *p* < 0.05 level, as indicated by the left-pointing arrow.^‡^Indicates a significant difference in PAL between boys and girls at a *p* < 0.05 level (*N* = 301).

The results of the univariate analysis of variance, shown in Figure 1, revealed a significant effect of organized sport participation on MVPA level [*F*_(5, 292)_ = 4.534, *p* = 0.001, η^2^ = 0.072, 1-β = 0.971]. Further analyses showed that this effect was evident for both boys [*F*_(5, 143)_ = 4.534, *p* = 0.002] and girls [*F*_(5, 149)_ = 2.454, *p* = 0.036] separately. Furthermore, there was a significant interaction between level of participation in organized sport and sex [*F*_(1, 292)_ = 4.114, *p* = 0.043, η^2^ = 0.014, 1–β = 0.525].

Follow-up analyses with Bonferroni corrections revealed that MVPA level was significantly higher among adolescents participating 11 h per week or more in organized sport, compared to those who did not participate in organized sport (mean dif. = 32 MVPA, 95% confidence interval [CI] = 6.6 to 57.4, *p* < 0.01), those who participated 1–2 h per week in organized sport (mean dif. = 30.4 MVPA, 95% confidence interval [CI] = 3.2 to 57.6, *p* < 0.05), and those who participated 3–4 h per week in organized sport (mean dif. = 25.8 MVPA, 95% confidence interval [CI] = 0.2 to 51.4, *p* < 0.05). Furthermore, MVPA level was significantly higher among adolescents participating 8–10 h per week or more in organized sport, compared to those who did not participate in organized sport (mean dif. = 20.3 MVPA, 95% confidence interval [CI] = 3.8 to 36.7, *p* < 0.01). Follow-up analyses according to sex showed that, only at the 3–4 h level, boys participated significantly more than girls in organized sport [*F*_(1)_ = 8.4, *p* = 0.006]. There were no significant sex differences in the other groups (*p* > 0.008, Bonferroni corrections).

**Figure 2:**
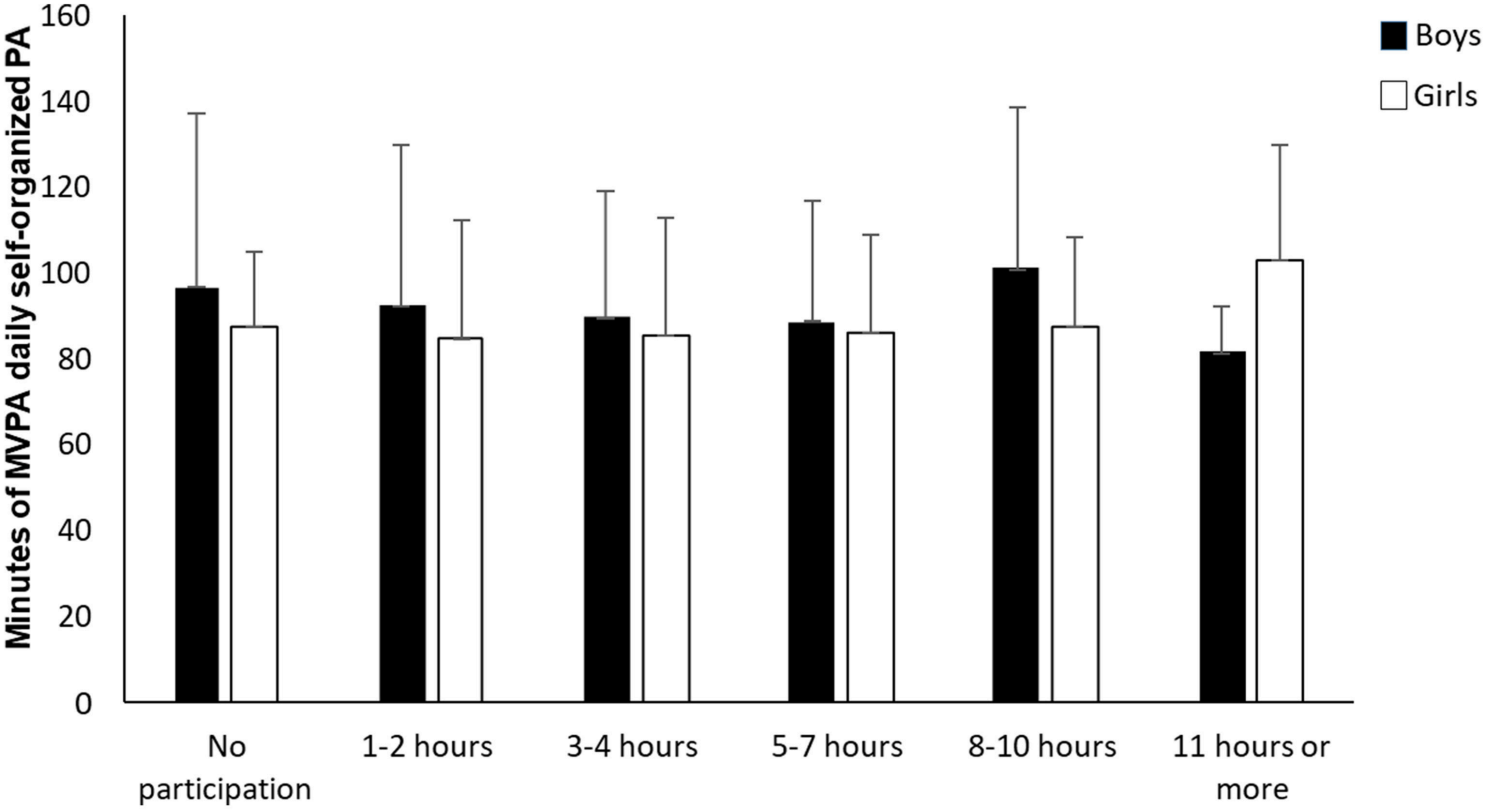
Differences in MVPA level among girls and boys according to increasing participation in self-organized physical activity (*N* = 301).

The results of the univariate analysis of variance, shown in Figure 2, revealed that different levels of participation in self-organized physical activity had no significant effect on adolescents' MVPA level [*F*_(5, 290)_ = 0.353, *p* = 0.880, η^2^ = 0.006, 1–β = 0.140]. Furthermore, there was no significant interaction between level of participation in self-organized physical activity and sex [*F*_(1, 290)_ = 0.298, *p* = 0.586, η^2^ = 0.001, 1–β = 0.085].

The Kruskal–Wallis H test showed that different levels of participation in organized sport significantly affected whether the adolescents fulfilled the recommended level of physical activity (X${}_{5}^{2}$ = 20.97, *p* < 0.001). This was also evident when analyzing boys only (X${}_{5}^{2}$ = 14.30, *p* = 0.014), but not when analyzing girls only (X${}_{5}^{2}$ = 10.90, *p* = 0.053). A follow-up test using Mann–Whitney U-test with Bonferroni corrections between the six groups identified significant differences between those participating <3 h in organized sport, and those participating 8 h or more (*p* < 0.008). There were no significant differences according to fulfillment of the recommended levels of physical activity between the other groups (*p* > 0.008).

The Kruskal–Wallis H test showed that the different levels of participation in self-organized physical activity did not significantly affect whether the adolescents fulfilled the recommended level of physical activity (X${}_{5}^{2}$ = 1.54, *p* = 0.908).

Figure 5 shows that, except for the group with no participation in organized sport (1–6), increased participation in self-organized physical activity did not increase adolescents' PAL. However, among adolescents that did not participate in organized sport, self-organized physical activity seemed to be of major importance.

## Discussion

The results in Figure 1 showed that the level of participation in organized sport was positively correlated with adolescents' overall PAL [medium effect according to Cohen, (32)]. The level of participation in self-organized physical activity, on the other hand (Figure 2), revealed no significant associations with adolescents' overall level of physical activity. These findings were strengthened by results showing that increased participation in self-organized physical activity did not increase the overall PAL of those who participated in organized sport (Figure 5). The importance of participating in organized sport on overall PAL is in line with previous research (13, 14, 22, 24, 26). Although it is difficult to explain the results categorically, one explanation could be that coaches organize their training with a higher intensity than adolescents do when they self-organize their physical activity.

Bélanger et al. (13) reported that adolescents involved in organized physical activity had a higher number of MVPA sessions per week than adolescents not involved in organized physical activities. However, Bélanger et al. (13) used self-reported PA-level data in their study, which may be less adequate than accelerometer data used in the present study. Accelerometers are objective measurements that decrease subjectivity and eliminate certain forms of bias, such as social desirability and recall problems (33). Participation in self-organized physical activity, on the other hand (Figure 2), showed no significant associations with adolescents' overall PAL (33, 34). The results in Figure 1 revealed that participating 8 h or more per week in organized sport resulted in significantly higher PAL than not participating at all, while participating 11 h or more per week in organized sport resulted in significantly higher PAL than participating <4 h per week. These findings contribute to a more nuanced understanding of the importance of participation level in organized sport.

The results in Figure 1 also revealed a significant interaction between level of participation in organized sport and sex on adolescents' activity level—showing that the level of participating in organized sport was of greater importance for boys than girls. Telford et al. (35) reported similar results from a study of Australian youth (8–16 years-old), for whom they found that participation in sports clubs led to three times as many steps, and significantly more minutes in MVPA per day for boys compared to girls. The finding is also consistent with Marques et al. (24), who found that among adolescents 10–18 years-old, the positive association between participation in organized sports and recommended levels of MVPA was only evident among boys and not among girls.

**Figure 3:**
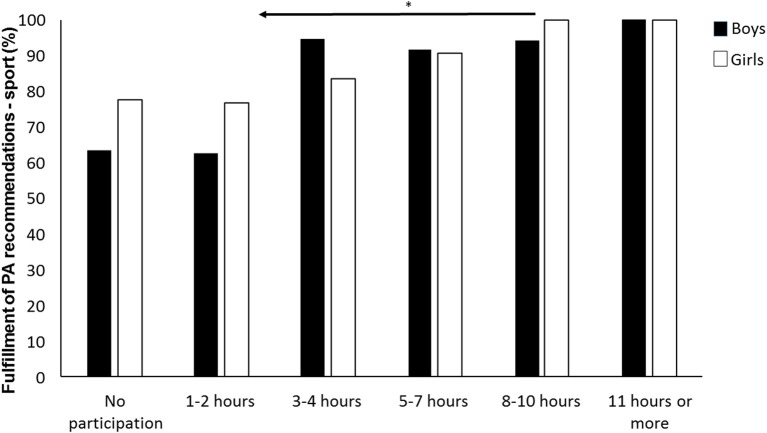
Differences in the fulfillment of health recommendations of boys and girls according to increasing participation in organized sport. ^*^Indicates a significant decrease in PAL between these groups and the groups left of the arrow at a *p* < 0.05 level, as indicated by the left-pointing arrow (*N* = 301).

Figure 3 showed that different levels of participation in organized sport significantly affected whether the adolescents met the recommended level of physical activity. This was also evident when analyzing boys only, but not when analyzing girls only. According to research that has demonstrated a positive association between participation in organized sport and recommended levels of MVPA (24, 35), this is not surprising. Figure 3 revealed that boys who participated <3 h per week (or not at all) in organized sport stood out with the lowest fulfillment of recommended physical activity levels. This indicates that adolescents who participate in organized sport constitute a heterogenic group. Consequently, a need exists for additional studies that explore adolescents' involvement in organized sport and MVPA on sub-group and individual levels. Socioeconomic variables should also be included here, because they can have a significant impact (21, 36–38).

**Figure 4:**
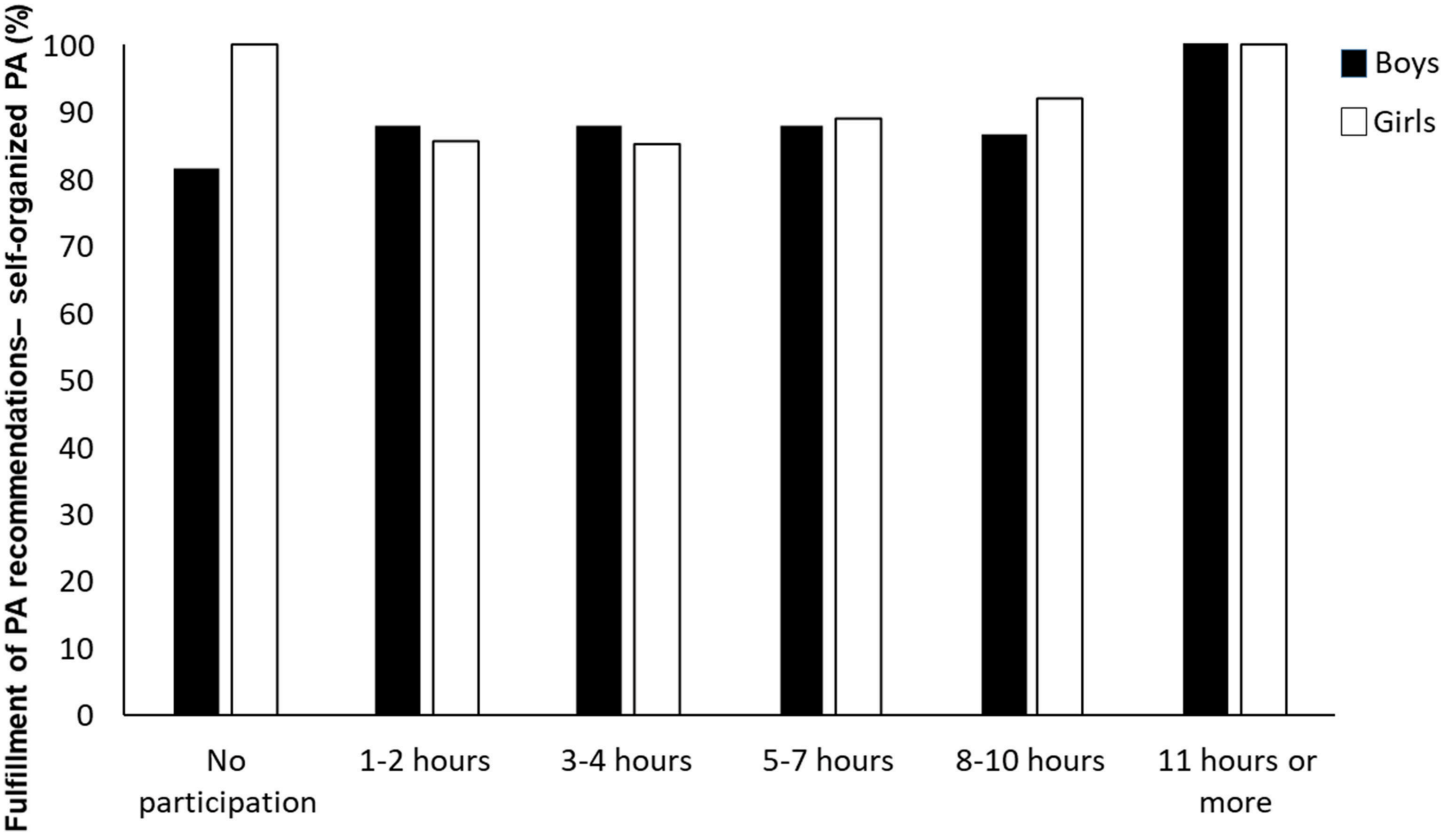
Differences in the fulfillment of recommended levels of physical activity of boys and girls according to increasing participation in self-organized physical activity (*N* = 301).

In contrast to participation in organized sport, different levels of participation in self-organized physical activity did not significantly affect whether the adolescents fulfilled the health recommendations of physical activity (Figure 4). As Dalene et al. (14) noted, different activities may yield varied levels of MVPA, and different individuals may accumulate various levels of MVPA during the same activities. The variations may even be more evident in self-organized physical activity settings, where individuals' opportunities to choose the intensity of their activity are greater than in organized settings.

The proportion of adolescents who fulfilled the recommended level of physical activity was relatively high in this study (86.9%), compared to the latest nationally representative study, based on the same method, device, and protocol as we used (21). Kolle et al. found that 69.8% of the girls and 86.2% of 9-year-olds met recommendations; whereas, for 15-year-old boys and girls, the percentages were only 43.2 and 58.1%, respectively. However, the response rates in Kolle's study were 73.1 and 54.7% for 9-year-olds and 15-year-olds, respectively, leading to questions about sample representability, at least for the oldest group. Our data were collected during the spring, when adolescents may be more active than in the winter (39). Hence, the proportion may have been somewhat lower had data been collected during the winter. Kolle et al. (21) investigated seasonal differences in activity levels for Norwegian girls and boys, 9 and 15 years-old, but did not find any consistent patterns of PAL related to seasonality for the 9-year-olds. For the 15-year-olds, they did not detect any statistical differences in PAL related to season (21), but they did not collect data during winter months at all (December through February).

**Figure 5:**
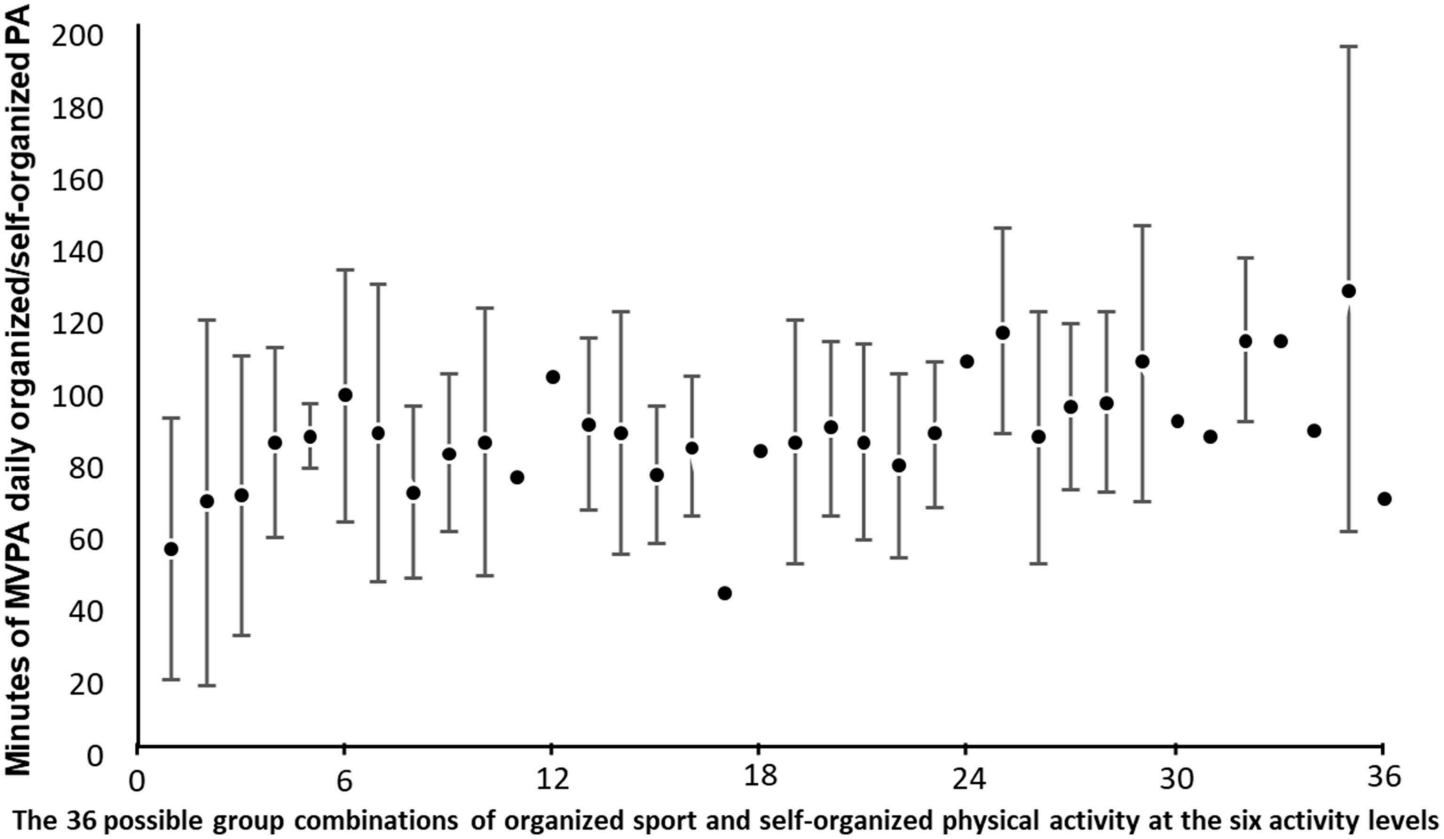
Differences in MVPA level among adolescents, according to their participation in both organized sport and self-organized physical activity. The figure is organized according to the 36 groups in Table 3, with six different levels of self-organized physical activity at each level of organized sport. Group 1–6 = no participation in organized sport (with increasing level of participation in self-organized physical activity); 7–12 = 1–2 h weekly in organized sport (with increasing level of participation in self-organized physical activity); 13–18 = 3–4 h weekly in organized sport (with increasing level of participation in self-organized physical activity); 19–24 = 5–7 h weekly in organized sport (with increasing level of participation in self-organized physical activity); 25–30 = 8–10 h weekly in organized sport (with increasing level of participation in self-organized physical activity); and 31–36 = 11 h or more weekly in organized sport (with increasing level of participation in self-organized physical activity) (*N* = 301). Standards deviations for some data points are absent because only one adolescent was available for that category.

Even if we found that participation in self-organized physical activity exhibited no significant associations with adolescents' overall level of physical activity, self-organized physical activity seems to be of major importance among adolescents who do not participate in organized sport, as shown in Figure 5. As participation in self-organized physical activity is not associated with fulfilling the recommended level of MVPA, one suggestion might be to investigate how to also engage these adolescents in organized sports. Pearce et al. (27) argued, however, that encouraging participation in organized physical activity in those who are more inactive may present a significant challenge, especially given limited investment in after-school sports. In addition, competitive sports-oriented opportunities do not suit some adolescents' preferences. Strategies to increase MVPA in the adolescent population require specific focus on organized leisure-time physical activity, especially outdoors, because their study showed that self-organized outdoor leisure time was associated with an increase in total daily MVPA of almost twice that of self-organized indoor leisure time (27). Peace et al. pointed to the importance of increasing the frequency of these sessions, maximizing the time that adolescents spend outdoors during unstructured leisure time, and developing environments or opportunities that facilitate greater MVPA participation once outdoors. It is appropriate to highlight that, although self-organized physical activity may not be associated with recommended levels of MVPA, it might still offer other benefits. For example, Tremblay and Willms (40) found a negative association between overweight and self-organized leisure-time activities, including both physical activities and other activities, such as art. They suggested that activities of lower intensities may assist to prevent negative behaviors related to sedentary lifestyles.

### Strengths and Limitations of the Study

The present study possesses several strengths. First, it includes a large number of participants, and reflects the actual distribution of boys and girls in Norwegian schools. Different types and sizes of schools from both rural and urban areas were also included in the study, which may provide a representative sample of Norwegian schools. Furthermore, the present study's use of accelerometry is based on a high-quality standard procedure that is not self-reported, and is validity- and reliability-tested for researching PAL for children (21, 41, 42). However, Rich et al. (30) point out that a lack of consensus exists regarding which intensity threshold to use, limiting the ability of researchers to make reliable comparisons of MVPA levels between studies. The use of an epoch length of 10 s also constitutes an advantage in this study, as it allows for inclusion of intermittent patterns of play (43). The International Children's Accelerometry Database (ICAD) uses 60-s epochs (44), but this is due to pooling data from older generation accelerometers and their storage capacity limitations (45), and does not reflect an optimal choice of method.

Nevertheless, the present study is not without limitations. Although accelerometry is considered to be the preferred measurement when assessing physical activity in free-living situations (46, 47), it underestimates activities related to cycling or riding vehicles (34), swimming and other water activities, which might lead to an underestimation of adolescents' overall PAL. However, according to swimming, 83% of the participants had not been to the swimming pool, and only 8% had been to the swimming pool twice or more. This implies that the proportion of adolescents in our sample actually fulfilling recommended physical activity levels may be even higher than 86.9%.

In the present study, each participant had to have at least 2 valid days to be included in the analyses, in accordance with other Norwegian studies (14, 21). In contrast, Cooper et al. (44) included only participants who provided at least 3 d of valid accelerometer data in their study of adolescents. However, Kolle et al. (21) did not find any significant differences in PAL between participants with two valid registration days and participants with 6–7 valid registration days. Furthermore, Rich et al. (30) also determined that a threshold of at least 2 d of valid PA data would provide a high reliability for subjects in studies of older primary school children. Furthermore, *post-hoc* analysis showed that the mean number of valid days in our study sample was 5.4 d (SD = 1.8). In sum, these findings lend support to the validity of our data.

When monitoring activity, there is always the risk that reactivity may affect the results. For instance, Foley, Beets and Cardinal (48) reported that during unrestricted play, children increase their activity when they know that they are being monitored. Although their sample was slightly younger than ours (aged 7–11 years), there is no reason to believe that young adolescents would exhibit lower rates of reactivity than children do. Ho et al. (49) indicated, for example, that adolescent girls (but not boys) increased their activity levels while being monitored. Moreover, attaching devices to measure PAL has been considered a component of behavior change strategies to promote and increase PA levels in adolescents (ibid), as a means of self-monitoring and awareness-raising. With the ActiGraph, adolescents are not informed about how many steps they achieve per day, but there may still be a “Hawthorne-like” effect of being monitored.

## Conclusion

The results of our study demonstrated that the level of participation in organized sport was positively associated with adolescents' overall PAL. It was also found that there was no significant association between time spent in self-organized physical activity, and adolescents' daily minutes of moderate and vigorous physical activity. Participating 11 h or more per week in organized sport resulted in significantly higher PAL than for adolescents participating < 4 h per week—a finding that contributes to a more nuanced understanding of the importance of participation level in organized sport. In addition, boys who participated < 3 h per week (or not at all) in organized sport, stood out with the lowest fulfillment of recommended PAL. Due to the health benefits of having a high PAL, our findings support previous research pointing to the critical importance of getting adolescents, especially boys, to participate in organized sport, and not drop out from organized sport during adolescence. Additional research is needed to elucidate the underlying mechanisms that seem to cause differences in the relative importance of participating in organized sport for girls and boys.

## Ethics Statement

The subjects were fully informed about the protocol prior to participating in the study. A written consent form was signed by the parents of the adolescents, and the youths themselves, according to accepted ethical research regulations. Approval to use the data and conduct the study was given by the Norwegian Center for Research Data (NSD).

## Author Contributions

PL contributed to the design, and writing the introduction, methods, results, discussion, and conclusion. HM contributed to the data collection and provided a critical review of the text during several revisions of the article. LI contributed to writing the introduction and discussion, as well as providing a critical review of all of the text during several revisions of the article and rewriting of the text. IL contributed to the design and provided a critical review of the text during several revisions of the article. CS contributed to writing the introduction and discussion, and provided a critical review of all of the text during several revisions of the article and rewriting of the text.

### Conflict of Interest Statement

The authors declare that the research was conducted in the absence of any commercial or financial relationships that could be construed as a potential conflict of interest.
